# Ethnic Differences in Family Childcare Providers' Nutrition- and Activity-Related Attitudes and Barriers

**DOI:** 10.1155/2021/6697006

**Published:** 2021-10-07

**Authors:** Qianxia Jiang, Alison Tovar, Patricia M. Risica, Kristen Cooksey Stowers, Marlene Schwartz, Caitlin Lombardi, Augustine Kang, Noereem Z. Mena, Kim M. Gans

**Affiliations:** ^1^Department of Human Development and Family Sciences, University of Connecticut, Storrs, CT, USA; ^2^Department of Nutrition and Food Sciences, University of Rhode Island, Kingston, RI, USA; ^3^Department of Behavioral and Social Health Sciences, Brown University School of Public Health, Providence, RI, USA; ^4^Center for Health Promotion and Health Equity, Brown University School of Public Health, Providence, RI, USA; ^5^University of Connecticut Institution for Collaboration in Health, Interventions and Policy, Storrs, CT, USA; ^6^Department of Allied Health Sciences, University of Connecticut, Storrs, CT, USA; ^7^University of Connecticut Rudd Center for Food Policy and Obesity, Hartford, CT, USA; ^8^Food Science and Human Nutrition Department, Colorado State University, Fort Collins, CO, USA

## Abstract

**Objective:**

The aim of the study is to examine family childcare providers' (FCCPs) attitudes and perceived barriers related to nutrition, physical activity (PA), and screen time (ST) behaviors of preschool children, exploring differences by provider ethnicity.

**Design:**

Baseline survey data from a cluster-randomized trial. *Participants*. Around 168 FCCPs completed a telephone survey, and 126 completed both telephone and in-person surveys. *Main Outcome Measures*. Phone and in-person surveys include 44 questions to assess FCCPs attitudes and perceived barriers regarding nutrition, PA, and ST in the family childcare home. *Analysis*. Associations by ethnicity (Latinx vs. non-Latinx) were assessed by ANOVA, adjusting for provider education and Bonferroni correction.

**Results:**

Some FCCP attitudes were consistent with national obesity prevention guidelines; for example, most FCCPs agreed that they have an important role in shaping children's eating and PA habits. However, many FCCPs agreed with allowing children to watch educational TV and did not agree that children should serve themselves at meals. Adjusting for education, there were statistically significant differences in attitude and perceived barrier scores by provider ethnicity. For example, Latinx FCCPs were more likely to agree that they should eat the same foods as children(*p* < .001) but less likely to agree that serving the food at meal and snack time is the adult's responsibility (*p* < .001). Latinx FCCPs were more like to perceive barriers related to children's safety playing outside (*p* < .001). *Conclusions and Implications*. While FCCPs hold some nutrition-, PA-, and ST-related attitudes consistent with national guidelines, training opportunities are needed for FCCPs to improve knowledge and skills and overcome perceived barriers related to nutrition and PA. Latinx FCCPs, in particular, may need culturally tailored training and support to overcome misperceptions and barriers.

## 1. Introduction

Childhood obesity, which has dramatically increased since 1988 [1], is a serious and urgent public health problem with substantial consequences for children's health, greater likelihood of obesity later in life, and long-term adverse health outcomes [2–5]. Children of low-income, racial/ethnic minority families are at a particularly high risk of overweight and obesity [6–8].

Early childhood is a critical time for developing eating patterns and food preferences, as well as physical activity (PA) and screen time (ST) behaviors, which may persist into adulthood [9–12]. However, eating, PA, and ST behaviors of preschoolers in the USA do not meet national guidelines [3, 12–14]. Thus, it is critical to understand the factors driving such behaviors in order to create effective interventions, environments, and policies to reduce childhood obesity [15, 16].

Although parents are important in shaping children's eating, PA, and ST behaviors, childcare providers also have an influential role [12, 17–19], as approximately 60% of preschool-aged children are in childcare [20]. Most obesity prevention research in childcare settings has been conducted in childcare centers, with much less research occurring in family childcare homes (FCCHs), the second most utilized non-relative childcare settings, which care for about 1.6 million US children [21, 22]. FCCHs provide care in a professional caregiver's home, where on average one caregiver cares for six children [23]. Compared to center-based childcare settings, FCCHs have different environments such as neighborhood-based home environments, flexible hours, and smaller groups of children [24]. FCCHs also have different regulatory standards for nutrition and PA, and providers there may face more challenges, such as limited resources, no support staff, and less business expertise [24]. Depending on the rules in different states, providers in FCCHs may or may not be required to be licensed [25]. Licensed providers are required to follow basic health and safety requirements [25]. In the United States, the common reasons why Latinx often get involved in family childcare homes include language barriers, gender ideologies of motherhood, the childrearing values of the dominant culture, providing economic support for their family, and new multiculturalism in the United States [26]. FCCHs are also utilized at higher rates by low-income and Latinx families due to cultural preferences for family-like care and economic and occupational constraints requiring flexible hours and lower costs [27, 28]. However, there is evidence that children enrolled in FCCHs are more likely to be overweight or obese than children in center-based care [18, 29]. Research also suggests that family childcare providers (FCCPs) may not always meet evidence-based nutrition and PA practices guidelines [18, 30–32].

In order to influence the nutrition, PA, and ST environments in FCCHs, it is important to discern the underlying psychosocial determinants, such as attitudes and perceived barriers that drive nutrition and activity-related practices of FCCPs. Furthermore, it is important to assess differences by provider ethnicity [6–8] because qualitative, survey, and observational data indicate potential differences in nutrition- and PA-related attitudes and practices by provider ethnicity [8, 30, 33–37], and studies examining children's health behaviors have shown ethnic differences [38–40]. Therefore, the purpose of this paper is to examine FCCPs' reported attitudes and perceived barriers related to nutrition, PA, and ST practices in their FCCHs, overall and by provider ethnicity (Latinx vs. non-Latinx). We hypothesize that for some attitudes and perceived barriers, the agreement scores of Latinx and non-Latinx providers will be significantly different.

## 2. Methods

The current study utilized baseline data from a cluster-randomized trial, Healthy Start/Comienzos Sanos, that is evaluating the efficacy of a multicomponent intervention to improve the food, PA, and ST environments of FCCHs, as well as the diet, PA, and sedentary behaviors of the 2- to 5-year-old children in their care . The intervention included tailored feedback, peer education, tailored newsletters and videos, active play toys, and group meetings with other FCCPs and peer coaches. Baseline data were collected from November 2015 to July 2018. Details about study recruitment, intervention, and evaluation were discussed elsewhere [41].

To be eligible for the study, participants had to operate an FCCH within 60 miles of Providence, RI; to be in operation for at least 6 months with plans to remain in operation for at least 1 year; and not closed for more than four weeks. The provider had to read and speak Spanish or English and care for at least one child between the ages of 2 and 5 years (who was not their own child or grandchild) for at least 10 hours per week who ate at least one meal and snack a day at the FCCH. Eligible providers completed a 30 minute baseline telephone survey followed by a 30 minute in-person survey at the FCCH and then received a $25 gift card. Other study measures followed, but they are not relevant to the current analysis. Surveys were administered by four research assistants with more than 10 years of research experience and extensive training and experience in computer-assisted survey interviewing. A total of 229 FCCPs were registered in our study. Of them, 169 were eligible to participate in our study. And one of them was no longer running FCCH. In the current study, 168 FCCPs completed the baseline telephone survey; 126 of these providers went on to complete the in-person survey. The Institutional Review Board of Brown University and University of Connecticut approved all study procedures.

### 2.1. Measures Relevant to the Current Analysis

#### 2.1.1. Demographics and Other Provider Characteristics

Providers' gender, ethnicity, and race were assessed during the telephone survey, with the following variables assessed during the in-person survey: age, household income, marital status, education, years in the USA, country of origin, years as a childcare professional, number of children currently in their care (and how many are their own children or grandchildren), and whether the FCCH accepts Child and Adult Care Food Program (CACFP) benefits (see Table 1).

#### 2.1.2. Attitudes and Perceived Barriers

The phone survey included 12 questions to assess FCCPs' attitudes about nutrition, PA, and ST in the FCCH setting. These questions included a series of statements modified from the validated Child Care Provider Healthy Eating and Activity Survey (Cronbach *⍺* = 0.72) [42], a statewide survey of childcare providers [34], and themes that emerged from formative research [35]. The in-person survey included an additional 32 questions on provider attitudes and perceived barriers about nutrition, PA, and ST in the childcare setting. These items were derived from previous research projects [43, 44], a review of relevant literature, and issues identified during formative research [35]. All questions were deemed to have content validity by study investigators, consultants, and a community advisory board. In addition, questions underwent cognitive assessment with a sample of FCCPs prior to the trial [41] (see Supplementary Material for the full list of questions).

For all questions, FCCPs were asked to express their level of agreement on a 5-point scale, and responses were scored as: agree a lot (5), agree a little (4), neither agree nor disagree (3), disagree a little (2), and disagree a lot (1). Examples of attitude questions included: “The children like the taste of skim or low-fat (1%) milk” and “You know how to help the children be more physically active.” For the purpose of presenting and discussing results, attitudes were characterized into “positive nutrition attitudes” and “misconceptions,” with attitudes that correspond to the Nutrition and Physical Activity Self-Assessment for Child Care (NAP SACC) [45–47] evidence-based guidelines for childcare practices being labeled as “positive nutrition attitudes” and “misconceptions” being those that do not correspond with these best practices (see Table 2 for a list of relevant NAPSACC best practices). Examples of perceived barrier questions included: “You are concerned about wasting food because the children won't eat healthy foods” and “You worry about children's safety when they play outside” (see Table 3 for nutrition-related attitudes and perceived barriers and Table 4 for PA- and ST-related attitudes and perceived barriers).

### 2.2. Statistical Analysis

Using the response scores, we calculated mean scores for each question and then examined associations by ethnicity (Latinx vs. Non-Latinx) using Chi-square test and ANOVA depending on the variable type. We then used ANOVA to assess ethnic differences in provider-reported attitudes and perceived barriers adjusting for FCCP education. The Bonferroni correction [48] was used to control the multiple comparisons, and the adjusted critical value was 0.0011. We ran all analyses using SPSS version 22 [49].

## 3. Results

The FCCP's demographic characteristics are presented in Table 1. FCCPs were all-female, and the majority were Latinx. The average provider age was 49 years; 11% of providers had no high school education. The lowest income providers represented 14% of the sample, and 72% were married or living with a partner. More than a third cared for their own children or grandchildren in their FCCHs. Over 80% participated in CACFP. FCCPs had an average of 7.5 children in their care (including their own children or grandchildren) and worked in the early childcare profession for 13 years. Compared to non-Latinx providers, Latinx providers were more likely to identify as a Black race; had lower education levels; had lower income; lived in the USA for fewer years; had fewer children in their care; and worked in the early childcare profession for fewer years. In addition, more than half of Latinx providers (61.2%) were Dominicans, 12.4% Colombians, 8.3% Puerto Ricans, and 5.8% Guatemalans.

### 3.1. Nutrition-Related Attitudes and Perceived Barriers (Table 3)

#### 3.1.1. Positive Nutrition Attitudes

Positive nutrition attitudes scores are presented in order of highest to lowest with higher scores indicating higher agreement. Three statements had scores above 4.0 indicating strong agreement by FCCPs: it is important for childcare providers to sit with children while they eat; it is important for childcare providers to eat the same food as the children; and childcare settings affect children's lifelong eating habits. Two statements had scored less than 3.0 indicating more disagreement than agreement: if the juice is limited, children will get enough vitamins, and children will take the right amount if you let them decide how much to eat. The rest of the statements had scores between 3.0 and 4.0 indicating more agreement than disagreement. Some differences by ethnicity were identified. After adjusting for education and multiple comparisons, Latinx FCCPs were more likely than non-Latinx FCCPs to agree that childcare providers should eat the same food as the children in their care.

#### 3.1.2. Nutrition-Related Misconceptions

Misconception scores are presented in order of highest to lowest with higher scores indicating more agreement with the misconception statements. Only two statements scored higher than a 3.0 indicating slightly more agreement than disagreement: society has gone overboard limiting sweets and other desirable food, and serving the food at meal and snack time is the adult's responsibility. Two statements had scored under 3.0 indicating more disagreement than agreement: how children eat while at childcare has little or no effect on food habits because those are formed at home, and giving children a food treat to reward good behavior is an effective way to manage their behavior. After adjusting for education and multiple comparisons, Latinx FCCPs were significantly more likely to agree with the following misconceptions than non-Latinx FCCPs: serving the food at meal and snack is the adult's responsibility (*p* < .001), and when children serve themselves, they are likely to eat less (*p* < .001).

#### 3.1.3. Nutrition-Related Perceived Barriers

FCCPs mainly disagreed with the barrier statements as mean scores were all less than 3.0. After adjusting for education and multiple comparisons, there were no positive provider-reported perceived barriers differed by ethnicity.

### 3.2. Physical Activity and Screen-Time-Related Attitudes and Perceived Barriers (Table 4)

Positive PA and ST attitudes are presented in order of highest to lowest with higher scores indicating higher agreement. Three statements had scores above 4.0 indicating strong agreement by FCCPs: you enjoy joining in with the children in play; children behave better when they are given plenty of PA; and childcare settings affect children's lifelong PA habits. Two statements had scored lower than 3.0 indicating higher levels of disagreement: parents send the right clothing for children to play outside, and parents want children to go outside even when it is cold or raining. The rest of the statements had scores between 3.0 and 4.0 indicating more agreement than disagreement. After adjusting for education and multiple comparisons, there were no positive provider-reported attitudes differed by ethnicity.

#### 3.2.1. ST Misconceptions

For the statement that “it is OK to let children watch educational programs on TV or the internet,” the mean score was 4.0 indicating that more providers agreed than disagreed with this statement. There were no differences by ethnicity.

#### 3.2.2. PA- and ST-Related Perceived Barriers

The highest mean barrier score (3.0) was for worrying about children's safety when they are playing outside indicating neither agreement nor disagreement overall. FCCPs mainly disagreed with the rest of the barrier statements as mean scores were 2.0 or less. Adjusting for education and multiple comparisons, Latinx FCCPs were more likely to agree with the following perceived barrier than non-Latinx FCCPs: you worry about the children's safety when they are playing outside (*p* < .001).

## 4. Discussion

To date, there are few studies examining quantitative data on FCCP's attitudes and beliefs related to childcare practices, and none to our knowledge examines differences by ethnicity. This study fills that research gap by examining FCCP-reported nutrition, PA- and ST-related attitudes, and perceived barriers and exploring differences between Latinx and non-Latinx providers. Similar to prior studies in center-based childcare settings [50–52] and qualitative studies in FCCHs [19, 53], we found that many FCCPs' attitudes were consistent with NAP SACC guidelines, but there were some misconceptions and perceived barriers. In addition, we did find some differences in FCCPs' attitudes and perceived barrier scores by ethnicity, which are discussed in more detail as follows.

### 4.1. Nutrition-Related Attitudes and Barriers

Most FCCPs believed that they have an important role in shaping children's lifelong eating habits. Qualitative research with Latinx FCCPs also supported this view with providers stating that they felt responsible for the health and well-being of the children they cared for and often felt as if they were a second parent [8, 35]. In the current study, most FCCPs also felt that they should sit with children at meals and eat the same foods. Latinx providers were more likely than non-Latinx providers to agree with the latter statement. A statewide survey with FCCPs found that more Latinx providers reported strongly agreeing to sitting with children during snacks and meals than did non-Latinx providers (80.0% vs. 59%; *p*=.02) [34]. Family meals are rooted in Latinx culture [54, 55], and qualitative research has indicated that Latinx FCCPs believe that they are an extension of the child's family [35]. However, another study found that Latinx providers in childcare centers and FCCHs were less likely to report sitting with children during meals, although these differences were not adjusted for education [36].

In the current study, a majority of FCCPs agreed that society has gone overboard limiting sweets and other desirable foods, which is in line with previous research demonstrating that FCCPs often do not meet best practice guidelines for limiting sugary foods [18, 30] and that current cultural and social norms in the US favor sweets [56]. In addition, in the current study, some FCCPs were concerned about children getting enough vitamins if they limited juice servings. In an observational study, 41% of FCCPs served more than the recommended amount (less than two 4–6 oz servings per week) of 100% fruit juice [30]. Other studies in FCCHs showed that more than half of the providers offered more than the recommended amount of 100% juice [24, 57]. FCCPs' fear of children not getting adequate nutrition could be a reason for the over-serving of juice.

The current study also found that many FCCPs had concerns about letting children serve themselves at meals even though NAP SACC guidelines recommend that children serve themselves to enhance self-regulation of eating [45–47]. For example, many FCCPs agreed that serving food at meal and snack time is the adult's responsibility, and few FCCPs agreed that if children served themselves they would take the right amount, with some providers agreeing that if children served themselves, they would eat less and make too much of a mess. Latinx FCCPs were more likely to feel the responsibility to serve food themselves and report concerns about children not eating enough. This is similar to prior research in both childcare centers and FCCHs, which found that Latinx providers were more likely to use controlling feeding practices during meals such as encouraging children to finish the food on their plate and pressuring children to eat more food than they wanted [18, 30, 34, 36, 58]. Similarly, qualitative research with Latinx FCCPs found that many providers were concerned that children may not eat enough food and would “help” the child eat by spoon-feeding them [35]. Latinx FCCPs shared the belief that children needed to be “strong and healthy” and that meant being “larger” [35], which is a common belief reported in Latinx culture [35, 59, 60] that may influence how Latinx FCCPs feed children.

In the current study, some FCCPs were concerned about wasting food when children served themselves. This concern is in line with qualitative research with FCCPs and research in head start childcare centers that identified the high cost of healthy food as a barrier [8, 61]. These concerns about wasting food and food costs may be due to the lower income levels of FCCPs.

### 4.2. Physical Activity and Screen-Time-Related Attitudes and Barriers

Regarding PA, most FCCPs believed that they have an important role in shaping children's PA habits, that they enjoy joining in children's play, and that children behave better when physically active, which is similar to previous research [24, 62]. While it is encouraging that FCCPs believe in the importance of PA and enjoy playing with children, other studies have shown that actual children's PA levels in FCCHs are low [63, 64]. One reason for this could be that as we found in the current study, many FCCPs worried about children's safety when they were playing outside, with more Latinx than non-Latinx FCCPs agreeing with this statement. In previous qualitative research, Latinx FCCPs also mentioned concerns for children's safety during PA [8, 35]. Latinx providers may be more likely to live in low-income neighborhoods that may be less safe in terms of crime and other built environment factors such as traffic and less green space [65]. Findings are consistent with previous research, which found that issues of access, availability, cost, and safety were major barriers to PA for low-income parents [66, 67].

In the current study, we found that many FCCPs agreed that it is OK to let children watch educational programs on TV or the Internet while NAP SACC guidelines recommend none or less than 30 minutes daily screen time in childcare [45–47]. This is consistent with findings from other studies that childcare providers rationalize the use of television/video/computer in childcare settings as an educational activity [18, 68–70]. However, studies have found that educational TV programs cannot compete with real-life activities and human interaction [71–74]. Diminished interaction can have negative effects on young children such as obesity [75], aggression [76], and decreased attention spans [73, 77]. Though none of these studies were conducted in childcare settings, social interactions as well as outdoor playtime may be displaced by television viewing in FCCHs [69]. Barriers to decreasing ST in FCCHs may include the need for FCCPs to use TV as a distraction while they prepare meals and the wide age ranges of children in FCCH; which may, for example, induce FCCPs to use ST with older children while care for younger children [8, 34, 35].

In previous research, FCCPs recognized the child's home environment as an important influence on children's PA and ST behaviors that can make it difficult for FCCP to engage the child in healthful activities [78, 79]. In the current study, many FCCPs agreed that parents do not want their children to go outside when it is cold or raining, which is consistent with qualitative research findings [8, 35]. In a qualitative study, FCCPs reported that parents did not want their children going outside if it was less than 50ºF [35], even though childcare regulations in many states expect children to go outside at much lower temperatures. FCCPs may be more apt to cater to parents' preferences rather than state guidelines because they are concerned about losing business [58].

This study does have some limitations that should be considered when interpreting the data. The study sample may not be representative of all FCCPs in greater Providence, RI, as participants were recruited for an intervention study, and the study purposely over-recruited Latinx FCCPs as they have largely been ignored in prior research. Because the FCCPs were enrolled in an intervention study, they could have been more interested in health, which may not be true of all providers in the area, so may lead to selection bias. While we adjusted for education, Latinx FCCPs were also more likely than non-Latinx providers to be born outside the USA, have fewer years in the childcare profession, and have lower income levels, so those disparities could also account for some of the differences in attitude and barrier scores. However, when adding these variables to the model, none of them was significant. Furthermore, we did not compare ethnicity based on Latin heritage due to the small sample size. The current analysis uses self-reported data rather than observations and thus could be subject to social desirability bias. The use of interviewer-administered surveys may also be more likely to yield socially desirable responses than self-administered surveys.

### 4.3. Implications for Research and Practice

While FCCPs have many positive nutrition- and activity-related attitudes to support obesity prevention, approaches are needed to further support FCCP efforts to improve knowledge and attitudes, reduce barriers, and foster best practices. Specifically, further education about appropriate beverages, screen time, feeding practices related to children's self-regulation, and communicating with parents about PA could be considered for FCCPs. Professional development offering opportunities to enhance knowledge of best practices in child nutrition, PA, and ST, as well as effective parental communication strategies to ensure consistent messages, may improve practices both in FCCHs and home environments [78].

As providers may influence the development of children's behaviors through role modeling and may not be confident in their own abilities to eat healthy and be physically active [8], future research could explore how training FCCPs may improve their own health behaviors [62] and their role modeling to children in their care [80, 81]. The current study also suggests that policy and environmental changes to create and improve safe public spaces, such as playgrounds, may benefit children's PA.

In the current study, Latinx providers who had fewer years in the childcare profession had more perceived barriers and more misconceptions than non-Latinx providers. Thus, less-experienced and Latinx providers may benefit from culturally tailored training and support. Future research is needed to further explore the role that FCCPs' ethnicity plays in shaping attitudes and perceived barriers. Moreover, it is important to study whether FCCP attitudes and perceived barriers are related to their nutrition and activity practices, as well as children's diet, PA, and ST in the FCCH. This research has the potential to inform interventions seeking to alter FCCPs' attitudes and perceived barriers, as well as their nutrition- and activity-related practices, and ultimately children's diet, PA, and ST behaviors and body mass index.

## Figures and Tables

**Table 1:** Family childcare providers' demographics by ethnicity.

Variable	Category	All, % (n)	Latinx^a^, % (n)	Non-Latinx^a^, % (n)	*p* value
Total sample			72	28	
Gender^a^ (*n* = 168)	Female	100 (168)	(121)	(47)	NA
Mean age^b^ (*n* = 126)		48.8	49.6	47.2	0.185
Provider's race^a^ (*n* = 168)					<0.001
	American Indian/Alaska native	4.8 (8)	6.6 (8)	0 (0)	
	Black/African American	13.7 (23)	14.0 (17)	12.8 (6)	
	Native Hawaiian/Pacific Islander	2.4 (4)	2.5 (3)	2.1 (1)	
	White/Caucasian	36.9 (62)	21.5 (26)	76.6 (36)	
	Others	28.0 (47)	37.2 (45)	4.3 (2)	
	Unknown/more than one	14.3 (24)	18.2 (22)	4.3 (2)	
Which of the following best describes your level of education?^b^ (*n* = 126)					0.021
	No high school diploma or general educational development	11.1 (14)	15.3 (13)	2.4 (1)	
	High school grad or general educational development	32.5 (41)	36.5 (31)	24.4 (10)	
	Associate's degree	38.1 (48)	35.3 (30)	43.9 (18)	
	Bachelor's degree	15.1 (19)	9.4 (8)	26.8 (11)	
	Master's degree or higher	3.2 (4)	3.5 (3)	2.4 (1)	
What is your total yearly household income from all sources?^b^ (*n* = 122)					<0.001
	Less than $25,000	13.9 (17)	19.3 (16)	2.6 (1)	
	$25,001–$50,000	50.0 (61)	60.2 (50)	28.2 (11)	
	$50,001–$75,000	20.5 (25)	16.9 (14)	28.2 (11)	
	$75,001–$100,000	9.8 (12)	3.6 (3)	23.1 (9)	
	$100,001 or more	5.7 (7)	0 (0)	17.9 (7)	
What country were you born in?^b^ (*n* = 126)					<0.001
	USA	28.6 (36)	7.1 (6)	73.2 (30)	
	Others	71.4 (90)	92.9 (79)	26.8 (11)	
What is your marital status?^b^ (*n* = 126)					0.363
	Single, never married	11.1 (14)	12.9 (11)	7.3 (3)	
	Married or living with a partner	72.2 (91)	68.2 (58)	80.5 (33)	
	Divorced	8.7 (11)	8.2 (7)	9.8 (4)	
	Separated	4.8 (6)	7.1 (6)	0 (0)	
	Widowed	3.2 (4)	3.5 (3)	2.4 (1)	
How many of those enrolled children are your own children or grandchildren?^b^ (*n* = 126)					0.651
	0	64.3 (81)	65.9 (56)	61 (25)	
	1	19.0 (24)	18.8 (16)	19.5 (8)	
	2	12.7 (16)	11.8 (10)	14.6 (6)	
	3	3.2 (4)	3.5 (3)	2.4 (1)	
	4	0.8 (1)	0 (0)	2.4 (1)	
Does your childcare home accept the child and adult care food program (CACFP) subsidies?^b^ (*n* = 126)					0.812
	Yes	81.7 (103)	81.2 (69)	82.9 (34)	
	No	18.3 (23)	18.8 (16)	17.1 (7)	
Mean years lived in the USA^b^		23.4	22.6	29.6	0.021
Mean number of children in family childcare homes^b^ (range: 1–16; median: 7)		7.5	6.9	8.6	0.004
Mean years working in early childcare profession^b^		12.7	11.1	16.2	<0.001

^a^Phone survey. ^b^In-person survey.

**Table 2:** Relevant best practices from nutrition and physical activity self-assessment for child care (NAPSACC) [45–47].

Domain	Best practices
Water	(i) Make drinking water available for children at all times
	(ii) Prompt children to drink water during each indoor and outdoor playtime

Juice	(i) Limit 100% fruit juice to no more than two 4–6 oz servings per week
	(ii) Only serve 100% fruit juice that has no sugar added

Milk	(i) Children of ages 2 and older should only be served skim or 1% milk
	(ii) Never serve flavored milk (milk with chocolate or strawberry syrup or with added sugar)

Vegetables	(i) Offer children vegetables two or more times a day
Fruit	(i) Offer children fruit two or more times a day
Whole grains	(i) Offer children high-fiber, whole grain foods two or more times a day
Snack foods	(i) Limit offering children sugary, salty, or fatty foods to less than 1 time per week or never
Mealtime environment	(i) Always sit at the table and eat with the children
	(ii) Teach children how to serve themselves or, in the case of older children, allow them to serve themselves

Self-regulation	(i) Always ask children if they are full before removing an unfinished meal or snack plate
	(ii) Always ask children if they are hungry before serving more food
	(iii) Never pressure children to eat more food than they want
	(iv) Do not use food or sweets as a reward or reward children for finishing their plate

Role modeling	(i) Enthusiastically role model eating and drinking healthy foods
Encouragement	(i) Always prompt and praise children for trying new or less preferred foods
Nutrition education	(i) Talk with children informally about nutrition and healthy eating as often as possible
Physical activity	(i) Provide children with ≥90 minutes of PA each day
Outdoor play	(i) Provide children with ≥60 minutes of outdoor play each day
Adult-led physical activity	(i) Provide children with ≥45 minutes of adult-led PA each day
Physical activity education	(i) Lead ≥1 planned PA lesson each week
Screen time	(i) Limit screen time to < 30 minutes or none per week
Participate in indoor PA with kids	(i) Always participate in indoor PA with children
Participate in outdoor PA with kids	(i) Always participate in outdoor PA with children
Parent communication PA	(i) Provide families with information on children's physical activity

^1^ Ammerman AS, Ward DS, Benjamin SE, et al. An intervention to promote healthy weight: Nutrition and Physical Activity Self-Assessment for Child Care (NAP SACC) theory and design. *Prev Chronic Dis*. 2007; 4 (3):A67. doi:A67 [pii], ^2^ Benjamin SE, Ammerman A, Sommers J, Dodds J, Neelon B, Ward DS. Nutrition and Physical Activity Self-assessment for Child Care (NAP SACC): Results from a Pilot Intervention {A figure is presented}. *J Nutr Educ Behav*. 2007; 39 (3): 142–149. doi:10.1016/j.jneb.2006.08.027, ^3^ Benjamin SE, Neelon B, Ball SC, Bangdiwala SI, Ammerman AS, Ward DS. Reliability and Benjamin SE, Neelon B, Ball SC, Bangdiwala SI, Ammerman AS, Ward DS. Reliability and validity of a nutrition and physical activity environmental self-assessment for child care. *Int J Behav Nutr Phys Act*. 2007;4(1):29. doi: 10.1186/1479-5868-4-29 m.

**Table 3:** Family childcare providers' nutrition-related attitudes and barriers by ethnicity status.

Variable	Mean (SD)			*p*	Adjusted^c^	
	All	Latinx	Non-Latinx		*F*	*P*
Positive attitudes						
It is important for childcare providers to sit with children while they eat^a^	4.85 (0.59)	4.88 (0.54)	4.79 (0.69)	0.379	0.06	0.812
Childcare providers should eat the same food as the children in their care^a^	4.65 (0.86)	4.83 (0.64)	4.19 (1.14)	<0.001	13.66	<0.001*∗*
Childcare settings affect children's lifelong eating habits^a^	4.47 (0.89)	4.50 (0.89)	4.40 (0.90)	0.550	0.94	0.335
You like the taste of the healthy food that the children are supposed to eat^b^	3.54 (2.17)	3.33 (2.27)	4.06 (1.82)	0.049	0.25	0.615
You know how to encourage the children to try new foods^b^	3.53 (2.14)	3.31 (2.25)	4.11 (1.72)	0.029	0.06	0.809
You know how to talk to children about healthy eating^b^	3.51 (2.12)	3.24 (2.22)	4.19 (1.66)	0.008	2.39	0.124
You have enough time to prepare healthy food as often as you would like^b^	3.42 (2.18)	3.19 (2.29)	4.00 (1.77)	0.030	0.05	0.827
You have enough time lead lessons about nutrition^b^	3.35 (2.09)	3.13 (2.19)	3.91 (1.70)	0.029	0.01	0.918
You know how to find materials to use to teach children about nutrition^b^	3.32 (2.13)	3.09 (2.22)	3.89 (1.76)	0.028	0.08	0.775
You have enough time to sit at the table with the children at meal and snack times^b^	3.18 (2.16)	3.02 (2.24)	3.62 (1.87)	0.106	0.05	0.832
If water was the only drink that you offered during playtime, the children would drink enough^b^	3.17 (2.22)	2.76 (2.26)	4.21 (1.74)	<0.001	9.63	0.002
The children like the taste of skim or low-fat (1%) milk^b^	3.07 (2.19)	2.74 (2.24)	3.94 (1.81)	0.001	7.12	0.009
Some dishes you make would taste just as good if you made them with whole grains^b^	3.04 (2.09)	2.72 (2.15)	3.85 (1.72)	0.001	5.674	0.019
If you were to limit the amount of 100% pure fruit juice the children drink, they would get enough vitamins^b^	2.96 (2.20)	2.70 (2.25)	3.64 (1.93)	0.013	1.55	0.215
If you let the children decide how much to eat, they will take the right amount^b^	2.04 (1.82)	1.80 (1.81)	2.64 (1.74)	0.007	2.33	0.130
Misconceptions						
Society has gone overboard limiting sweets and other desirable food^a^	3.17 (1.65)	3.25 (1.68)	2.98 (1.57)	0.344	1.78	0.185
Serving the food at meal and snack time is the adult's responsibility^b^	3.07 (2.14)	3.12 (2.25)	2.96 (1.85)	0.668	16.39	<0.001*∗*
When children serve themselves, they are likely to eat less^a^	3.01 (1.47)	3.36 (1.37)	2.13 (1.36)	<0.001	27.55	<0.001*∗*
How children eat while at childcare has little or no effect on food habits because those are formed at home^a^	2.82 (1.66)	3.17 (1.64)	1.91 (1.37)	<0.001	2.42	0.123
Giving children a food treat to reward good behavior is an effective way to manage their behavior^a^	1.89 (1.37)	2.06 (1.46)	1.47 (0.10)	0.012	3.18	0.077
Perceived barriers						
If you let the children serve themselves, they will make too much of a mess^b^	2.54 (2.01)	2.54 (2.08)	2.53 (1.84)	0.988	4.27	0.041
The children eat unhealthy foods at home, so it is hard to get them to eat healthy foods in your care^b^	2.44 (2.03)	2.34 (2.10)	2.70 (1.82)	0.298	0.23	0.630
If you let the children serve themselves, they will waste too much food^b^	2.36 (1.90)	2.28 (1.96)	2.57 (1.77)	0.371	0.40	0.531
Fresh fruits and vegetables go bad too quickly to be able to serve them very often^b^	2.15 (2.00)	2.21 (2.07)	2.02 (1.81)	0.591	4.49	0.036
It is hard to serve healthy foods because the children are picky^b^	2.13 (1.93)	2.05 (1.99)	2.32 (1.80)	0.419	0.32	0.576
You are concerned about wasting food because the children will not eat healthy foods^b^	1.85 (1.83)	1.91 (1.98)	1.70 (1.40)	0.512	5.38	0.022
Fresh fruits and vegetables are too expensive to serve as often as you would like^b^	1.72 (1.75)	1.72 (1.82)	1.72 (1.57)	0.988	2.86	0.093

*Note.* Higher scores indicate higher agreement with the statement. ^a^Phone survey, ^b^in-person survey, and ^c^ANOVA with family childcare providers' education as a covariate. The Bonferroni correction was used to control the multiple comparisons, and the adjusted critical value was 0.0011.

**Table 4:** Family childcare providers' physical activity and screen-time-related attitudes and barriers by ethnicity status.

Variable	Mean (SD)			*p*	Adjusted^c^	
	All	Latinx	Non-Latinx		*F*	*P*
Positive attitudes						
You enjoy joining in with the children in play^a^	4.94 (0.26)	4.95 (0.22)	4.91 (0.35)	0.431	0.17	0.680
Children behave better when they are given plenty of physical activity^a^	4.85 (0.48)	4.83 (0.50)	4.89 (0.43)	0.414	0.01	0.922
Child care settings affect children's lifelong physical activity habits^a^	4.49 (0.99)	4.45 (1.06)	4.62 (0.74)	0.315	1.07	0.303
You have enough time to help the children be physically active^b^	3.68 (2.15)	3.45 (2.27)	4.30 (1.68)	0.021	0.30	0.588
You know how to help the children be more physically active^b^	3.52 (2.15)	3.23 (2.25)	4.28 (1.68)	0.004	5.05	0.026
You know how to get the children to be physically active during bad weather^b^	3.43 (2.12)	3.21 (2.21)	4.00 (1.77)	0.031	0.29	0.592
Parents feel it is safe for children to play outside^b^	3.36 (2.15)	3.05 (2.20)	4.17 (1.77)	0.002	3.35	0.069
You know how to lead physical activity lessons^b^	3.36 (2.13)	3.13 (2.19)	3.96 (1.83)	0.023	0.299	0.585
Parents send the right clothing for children to play outside^b^	2.83 (2.06)	2.86 (2.19)	2.77 (1.71)	0.793	7.52	0.007
Parents want children to go outside even when it's cold or raining^b^	1.64 (1.53)	1.48 (1.47)	2.04 (1.62)	0.031	0.28	0.599
Misconceptions						
It is OK to let children watch educational programs on TV or the Internet^a^	3.98 (0.93)	4.05 (0.90)	3.79 (0.98)	0.100	0.74	0.393
Perceived barriers						
You worry about the children's safety when they are playing outside^b^	3.05 (2.25)	3.41 (2.31)	2.13 (1.80)	0.001	112.42	<0.001*∗*
The children are not physically active at home, so it's hard to get them to be physically active in your care^b^	1.96 (1.83)	1.97 (1.88)	1.96 (1.72)	0.976	2.14	0.136
The children would rather watch TV or play video games than do physical activities^b^	1.79 (1.79)	1.90 (1.92)	1.51 (1.38)	0.206	7.94	0.006
You get too tired to join in active play with the children^b^	1.50 (1.51)	1.49 (1.60)	1.53 (1.27)	0.865	1.99	0.161
The children have a lot of screen time at home, so it is hard to limit their screen time in your care^b^	1.49 (1.55)	1.36 (2.51)	1.85 (1.63)	0.063	0.54	0.462

*Note.* Higher scores indicate higher agreement with the statement. ^a^Phone survey, ^b^in-person survey, and ^c^ANOVA with family childcare providers' education as a covariate. The Bonferroni correction was used to control the multiple comparisons and the adjusted critical value was 0.0011.

## Data Availability

The data that support the findings of this study are available on request from the corresponding author.

## Supplementary Materials

Supplementary Materials The full list of questions of FCCPs' attitudes about nutrition, PA, and ST in the FCCH setting is provided in the Supplementary Material. These questions included a series of statements modified from the validated Child Care Provider Healthy Eating and Activity Survey (Cronbach *⍺* = 0.72) [42], a statewide survey of childcare providers [34], and themes that emerged from formative research [35].
