# Supplementary material for: Ethnic Differences in Family Childcare Providers' Nutrition- and Activity-Related Attitudes and Barriers
Source: J Obes. 2021 Oct 7;2021:6697006. doi: 10.1155/2021/6697006 (PMC8516587; doi:10.1155/2021/6697006)
Supplement: Supplementary Materials — The full list of questions of FCCPs' attitudes about nutrition, PA, and ST in the FCCH setting is provided in the Supplementary Material. These questions included a series of statements modified from the validated Child Care Provider Healthy Eating and Activity Survey (Cronbach ⍺ = 0.72) [42], a statewide survey of childcare providers [34], and themes that emerged from formative research [35]. [file 6697006.f1.docx]

**Supplemental Files**

**Healthy Start study Attitude and Perceived Barrier Survey Questions**

Now I am going to make some statements and ask whether you agree or disagree with them and how strongly you agree or disagree. (agree a little, agree a lot, neither agree nor disagree, disagree a little, disagree a lot)

1. You enjoy joining in with the children in play. Do you agree, disagree, or neither? A little or a lot? (A)

2. Children behave better when they are given plenty of physical activity. Do you agree, disagree, or neither? A little or a lot? (A)

3. It is OK to let children watch educational programs on TV or the internet. Do you agree, disagree, or neither? A little or a lot? (A)

4. When children serve themselves, they are likely to eat less. Do you agree, disagree, or neither? A little or a lot? (A)

5. Giving children a food treat to reward good behavior is an effective way to manage their behavior. Do you agree, disagree, or neither? A little or a lot? (A)

6. Society has gone overboard limiting sweets and other desirable food. Do you agree, disagree, or neither? A little or a lot? (A)

7. Child care providers should eat the same food as the children in their care. Do you agree, disagree, or neither? A little or a lot? (A)

8. It is important for child care providers to sit with children while they eat. Do you agree, disagree, or neither? A little or a lot? (A)

9. How children eat while at child care has little or no effect on food habits because those are formed at home. Do you agree, disagree, or neither? A little or a lot? (A)

10. Child care settings affect children’s lifelong eating habits. Do you agree, disagree, or neither? A little or a lot? (A)

11. Child care settings affect children’s lifelong physical activity habits. Do you agree, disagree, or neither? A little or a lot? (A)

12. If water was the only drink that you offered during play time, the children would drink enough. Do you agree, disagree, or neither? A little or a lot? (A)

13. If you were to limit the amount of 100% pure fruit juice the children drink, they would get enough vitamins. Do you agree, disagree, or neither? A little or a lot? (A)

14. The children like the taste of skim or lowfat (1%) milk. Do you agree, disagree, or neither? A little or a lot? (A)

15. You have enough time to prepare healthy foods as often as you would like. Do you agree, disagree, or neither? A little or a lot? (A)

16. Fresh fruits and vegetables go bad too quickly to be able to serve them very often. Do you agree, disagree, or neither? A little or a lot? (PA)

17. Fresh fruits and vegetables are too expensive to serve as often as you would like. Do you agree, disagree, or neither? A little or a lot? (PA)

18. You are concerned about wasting food because the children won’t eat healthy foods. Do you agree, disagree, or neither? A little or a lot? (PA)

19. It is hard to serve healthy foods because the children are picky. Do you agree, disagree, or neither? A little or a lot? (PA)

20. Some dishes you make would taste just as good if you made them with whole grains. Do you agree, disagree, or neither? A little or a lot? (A)

21. You have enough time to sit at the table with the children at meal and snack times. Do you agree, disagree, or neither? A little or a lot? (A)

22. If you let the children serve themselves, they will make too much of a mess. Do you agree, disagree, or neither? A little or a lot? (PA)

23. If you let the children serve themselves, they will waste too much food. Do you agree, disagree, or neither? A little or a lot? (PA)

24. Serving the food at meal and snack time is the adult’s responsibility. Do you agree, disagree, or neither? A little or a lot? (A)

25. If you let the children decide how much to eat, they will take the right amount. Do you agree, disagree, or neither? A little or a lot? (A)

26. You like the taste of the healthy food that the children are supposed to eat. Do you agree, disagree, or neither? A little or a lot? (A)

27. You know how to encourage the children to try new foods. Do you agree, disagree, or neither? A little or a lot? (A)

28. You know how to talk to children about healthy eating. Do you agree, disagree, or neither? A little or a lot? (A)

29. You have enough time to lead lessons about nutrition. Do you agree, disagree, or neither? A little or a lot? (A)

30. You know how to find materials to use to teach children about nutrition. Do you agree, disagree, or neither? A little or a lot? (A)

31. You have enough time to help the children be physically active. Do you agree, disagree, or neither? A little or a lot? (A)

32. You know how to help the children be more physically active. Do you agree, disagree, or neither? A little or a lot? (A)

33. The children would rather watch TV or play videogames than do physical activities. Do you agree, disagree, or neither? A little or a lot? (PA)

34. You get too tired to join in active play with the children. Do you agree, disagree, or neither? A little or a lot? (PA)

35. You know how to get the children to be physically active during bad weather. Do you agree, disagree, or neither? A little or a lot? (A)

36. Parents send the right clothing for children to play outside. Do you agree, disagree, or neither? A little or a lot? (A)

37. Parents want children to go outside even when it’s cold or raining. Do you agree, disagree, or neither? A little or a lot? (A)

38. Parents feel it is safe for children to play outside. Do you agree, disagree, or neither? A little or a lot? (A)

39. You worry about the children’s safety when they are playing outside. Do you agree, disagree, or neither? A little or a lot? (PA)

40. You know how to lead physical activity lessons. Do you agree, disagree, or neither? A little or a lot? (A)

41. The children eat unhealthy foods at home, so it’s hard to get them to eat healthy foods in your care. Do you agree, disagree, or neither? A little or a lot? (PA)

42. The children are not physically active at home, so it’s hard to get them to be physically active in your care. Do you agree, disagree, or neither? A little or a lot? (PA)

43. The children have a lot of screen time at home, so it’s hard to limit their screen time in your care. Do you agree, disagree, or neither? A little or a lot? (PA)
